# Towards in vivo characterization of thyroid nodules suspicious for malignancy using multispectral optoacoustic tomography

**DOI:** 10.1007/s00259-023-06189-1

**Published:** 2023-04-11

**Authors:** Milou E. Noltes, Maximilian Bader, Madelon J. H. Metman, Jasper Vonk, Pieter J. Steinkamp, Jan Kukačka, Henriette E. Westerlaan, Rudi A. J. O. Dierckx, Bettien M. van Hemel, Adrienne H. Brouwers, Gooitzen M. van Dam, Dominik Jüstel, Vasilis Ntziachristos, Schelto Kruijff

**Affiliations:** 1 Department of Surgical Oncology, University of Groningen, University Medical Center Groningen, Groningen, the Netherlands; 2 Department of Nuclear Medicine and Molecular Imaging, University Medical Center Groningen, University of Groningen, Groningen, the Netherlands; 3 Chair of Biological Imaging at the Central Institute for Translational Cancer Research (TranslaTUM), School of Medicine, Technical University of Munich, Munich, Germany; 4 Institute of Biological and Medical Imaging, Helmholtz Zentrum München, Neuherberg, Germany; 5 Department of Oral and Maxillofacial Surgery, University Medical Center Groningen, University of Groningen, Groningen, the Netherlands; 6 Department of Radiology, University Medical Center Groningen, University of Groningen, University Medical Center Groningen, Groningen, the Netherlands; 7 Department of Pathology, University of Groningen, University Medical Center Groningen, Groningen, the Netherlands; 8 AxelaRx/TRACER Europe BV, Groningen, the Netherlands; 9 Institute of Computational Biology, Helmholtz Zentrum München, Neuherberg, Germany; 10 Munich Institute of Robotics and Machine Intelligence (MIRMI), Technical University of Munich, Munich, Germany

**Keywords:** Thyroid nodule, Thyroid cancer, Multispectral optoacoustic tomography, Optoacoustic, Photoacoustic

## Abstract

**Purpose:**

Patient-tailored management of thyroid nodules requires improved risk of malignancy stratification by accurate preoperative nodule assessment, aiming to personalize decisions concerning diagnostics and treatment. Here, we perform an exploratory pilot study to identify possible patterns on multispectral optoacoustic tomography (MSOT) for thyroid malignancy stratification. For the first time, we directly correlate MSOT images with histopathology data on a detailed level.

**Methods:**

We use recently enhanced data processing and image reconstruction methods for MSOT to provide next-level image quality by means of improved spatial resolution and spectral contrast. We examine optoacoustic features in thyroid nodules associated with vascular patterns and correlate these directly with reference histopathology.

**Results:**

Our methods show the ability to resolve blood vessels with diameters of 250 μm at depths of up to 2 cm. The vessel diameters derived on MSOT showed an excellent correlation (*R*^2^-score of 0.9426) with the vessel diameters on histopathology. Subsequently, we identify features of malignancy observable in MSOT, such as intranodular microvascularity and extrathyroidal extension verified by histopathology. Despite these promising features in selected patients, we could not determine statistically relevant differences between benign and malignant thyroid nodules based on mean oxygen saturation in thyroid nodules. Thus, we illustrate general imaging artifacts of the whole field of optoacoustic imaging that reduce image fidelity and distort spectral contrast, which impedes quantification of chromophore presence based on mean concentrations.

**Conclusion:**

We recommend examining optoacoustic features in addition to chromophore quantification to rank malignancy risk. We present optoacoustic images of thyroid nodules with the highest spatial resolution and spectral contrast to date, directly correlated to histopathology, pushing the clinical translation of MSOT.

**Supplementary Information:**

The online version contains supplementary material available at 10.1007/s00259-023-06189-1.

## Introduction

The incidence of thyroid nodules in clinical practice is high and is still increasing [1]. Thyroid nodules can be detected with high-resolution ultrasound in 19 to 68% of randomly selected individuals using high-resolution ultrasound (US) [2, 3]. The aim in thyroid nodule diagnostics is to assess the risk of malignancy, which occurs in 5 to 15% of cases [4–6].

International guidelines recommend the performance of a head and neck US for cancer risk stratification [7–9]. These US-based risk stratifications are used to identify nodules suspicious for malignancy and guide the need for fine-needle aspiration (FNA) cytology. Currently, cytology of FNA using the Bethesda System for Reporting on Thyroid Cytopathology (BSRTC) provides the most definitive diagnostic information (refer to Supplementary Table 1) [10]. Notwithstanding, 30% of all FNAs are classified as Bethesda I, III, or IV, indicating non-diagnostic, undetermined significance or follicular neoplasm [11]. In most cases, a diagnostic hemithyroidectomy (i.e., histopathology) is considered the appropriate choice rather than watchful waiting for a definitive diagnosis. Yet, 70–80% of these patients will have a benign diagnosis [11], implying significant overtreatment with risk of postoperative morbidity (e.g., parathyroid injury, hypothyroidism, and laryngeal nerve injury) that affects the quality of life of these patients [12–14].

Patient-tailored management of patients with thyroid nodules suspicious for malignancy would benefit significantly from improved cancer risk stratification by improving the accuracy of nodule assessment. Multispectral optoacoustic tomography (MSOT) is a rapidly growing imaging modality that may accommodate this need by adding molecular contrast to conventional US. MSOT enables non-invasive macroscopic imaging (i.e., at the organ level) at depths of several centimeters. By detecting US waves induced by pulsed laser light illumination, referred to as optoacoustic or photoacoustic waves, MSOT has the potential to distinguish and quantify different intrinsic tissue chromophores such as oxy- (HbO2), deoxyhemoglobin (HbR), and lipid and melanin, as well as exogenous contrast agents [15]. The clinical potential of MSOT for disease characterization has been shown in various diseases, including thyroid nodules [16–20], breast cancer [21], or Crohn’s disease [22]. The first studies imaging thyroid tissue with MSOT showed the potential to differentiate between malignant and normal tissue during ex vivo imaging [16, 18]. In vivo MSOT imaging performed on healthy volunteers showed that it was feasible to image the thyroid and identify vascular structures, which were validated using Doppler ultrasound [17]. While Ultrasound Doppler can visualize blood flow in lesions, it is not suitable to visualize blood flow with low velocity. In contrast, MSOT uses the much more distinctive optical absorption of HbO2 and HbR to visualize vessels. Microvasculature typically exhibits low-velocity blood flow, and previous studies suggest that optoacoustic imaging may be more effective than ultrasound Doppler [23]. Exactly these changes in microvasculature blood flow may be relevant for differentiating benign and malignant nodules [24]. Two MSOT studies confirmed the possibility of distinguishing between benign and malignant nodules and healthy thyroid tissue in vivo, and the latter proposed a strategy using optoacoustic imaging to reduce unnecessary FNA [19, 20]. The common result in all the studies on thyroid nodules was a decrease in hemoglobin oxygen saturation between malignant and benign nodules [16–20]. However, these studies’ findings might be distorted because simple data processing and inversion techniques, typically based on back-projection algorithms, were employed that contain fundamental limitations decreasing image quality and spectral unmixing accuracy [21]. In this exploratory pilot study, we present in vivo MSOT images of thyroids nodules suspicious for malignancy with next-level image quality and accuracy, i.e., spatial resolution and spectral contrast, utilizing recently developed enhanced data processing and image reconstruction methods [21, 25–27]. For the first time, we directly correlate MSOT images with histopathology data on a detailed level, the latter considered the gold standard confirming the high spatial resolution and spectral contrast of our images. We collect data from 38 thyroid nodules, analyze data sets from 27 thyroid nodules and present a detailed analysis of five representative cases to outline thyroid nodule features. Anatomical and optoacoustic features from selected regions of the image are provided. Despite these promising features in selected patients, we cannot reproduce results discriminating benign and malignant thyroid nodules based on mean oxygen saturation in the nodule according to previous publications [16–20]. Thus, we investigate general limitations and distorting effects that obstruct quantitative MSOT imaging to date. The findings presented herein provide, to the best of our knowledge, the highest quality optoacoustic images of thyroid nodules suspicious for malignancy to date.

## Methods

### Study design and patients

**Fig. 1:**
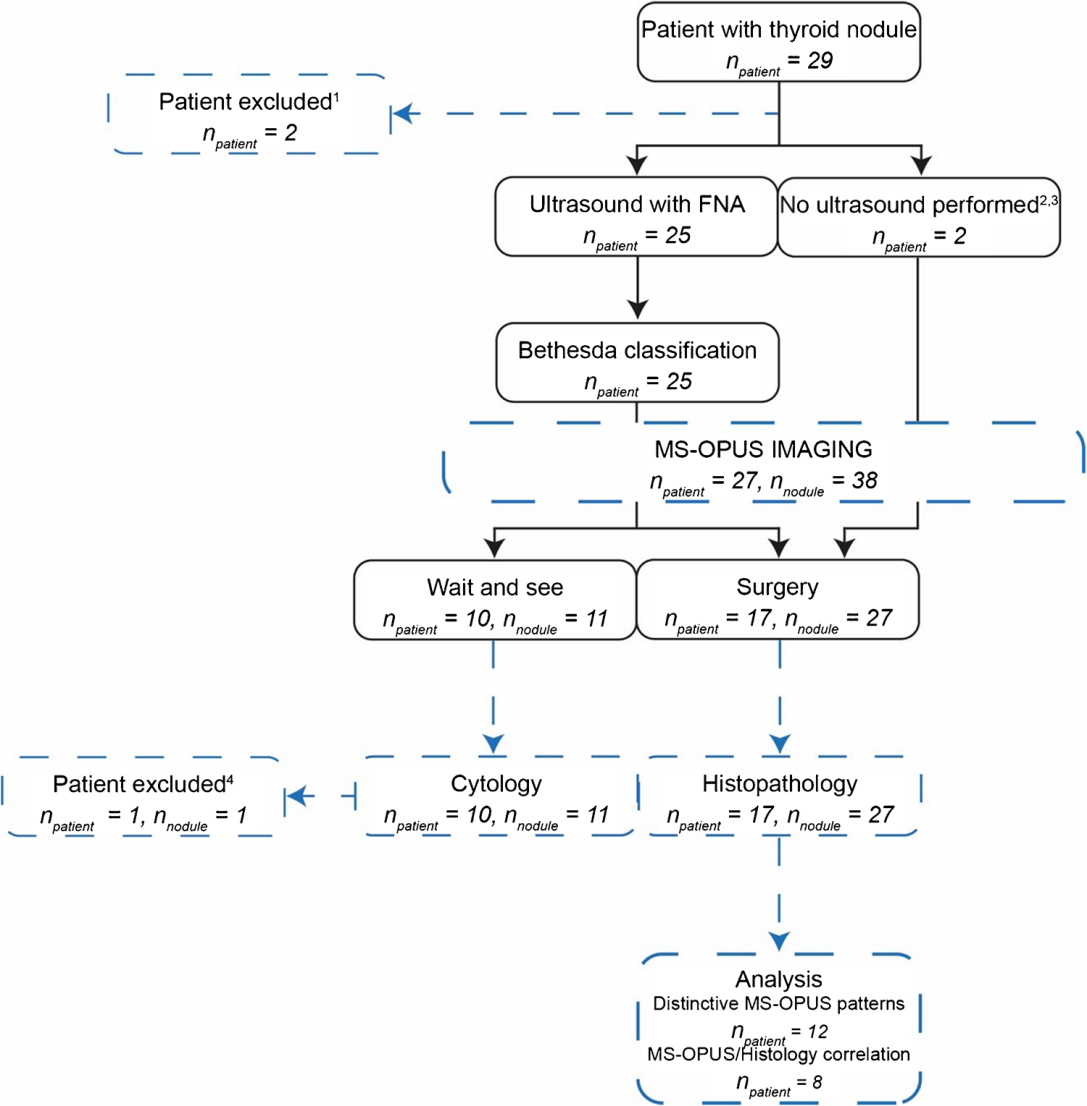
Study workflow including the numbers of included patients and imaged index nodules. Black lines represent the standard of care. The dashed blue lines represent the study intervention and the data included in the final analysis. ^1^ Two patients were excluded due to technical failure of the MSOT device. ^2^ Patient was scheduled for thyroidectomy after histologically proven distant metastasis of papillary thyroid cancer. ^3^ Patient was diagnosed with goiter causing obstructive symptoms of breathing or swallowing difficulties and was directly scheduled for thyroidectomy. ^4^ One patient was excluded from data analysis as this patient was still in follow-up for metastatic colorectal cancer (Bethesda 4). *n*_patient_ = number of patients included, FNA = fine needle aspiration, n_nodule_ = number of index nodules

The current study is a non-blinded, single-center, prospective, pilot study. Patients ≥ 18 years with thyroid nodules who underwent diagnostic US combined with FNA or had an indication for thyroidectomy (e.g., distant metastasis or goiter) were included between September 2020 and April 2021. An index nodule was defined as a nodule for which diagnostic imaging was indicated. Since this was a pilot study, patient inclusion was stopped after including 21 nodules with final histopathology (Fig. 1). Patients were excluded if they had previous surgery in the head and neck area on the ipsilateral side of the index nodule, received prior radiotherapy in the head and neck area, or were pregnant. All patients gave written informed consent before enrolment. The study was approved by the institutional review board Groningen and registered at ClinicalTrials.gov (NCT04730726). All procedures performed in studies involving human participants were in accordance with the ethical standards of the institutional and/or national research committee and with the 1964 Helsinki declaration and its later amendments or comparable ethical standards. Approval was granted by the Medical Ethics Committee of the University Medical Center Groningen (METc number METc 2019/371).

### MS-OPUS image acquisition

All MSOT procedures were performed using a clinical hybrid US–MSOT (MS-OPUS) system (MSOT Acuity Echo prototype; iThera Medical GmbH, Germany). This system uses a Nd:YAG laser (25-Hz repetition rate, 4-–7-ns pulse duration) for the emission of 25-mJ light pulses. A two-dimensional handheld concave detector array (256 transducer elements with a center frequency of 3.4 MHz and bandwidth (− 6 dB) of 60% in receive-transmit mode, 125° angular coverage) was used for cross-sectional imaging with a field of view of 40 × 40 mm and in-plane spatial resolution up to 200 µm [21, 28].

The thyroid nodule and contralateral healthy thyroid was imaged transversely with the patient in a supine position with the neck in hyperextension. The US signal was detected for anatomical guidance of the imaging procedure. At the position of interest, in average 20 MSOT images consisting of 14 optical wavelengths ranging from 680 to 1195 nm (680, 700, 730, 760, 800, 850, 900, 930, 970, 1000, 1030, 1064, 1100, and 1195 nm) have been acquired (acquisition time of single frame: 0.56 s). These wavelengths were chosen to enable detection and reliable unmixing of Hb, HbO2, lipids, H2O, and collagen in accordance with previous studies [29, 30]. Simultaneously, a co-registered US image was obtained with the MS-OPUS device. Per patient in average nine positions and orientations of interest of both healthy and diseased thyroid tissue were recorded. The number of imaged positions varied between patients depending on the number of thyroid nodules. In total, the imaging time per patient ranged from 5 to 10 min including the time to determine the correct position and probe orientation. Patients were asked to hold their breath during image acquisition at single selected positions of interest to reduce breathing motion. To later correlate the location and orientation of the chosen scans to histopathology, the operator notated, based on anatomical structures, the place of the probe during imaging based on anatomical structures. The segmentation of the thyroid nodule was performed with the knowledge of the nodule position based on previous clinical ultrasonography.

### Data processing, image reconstruction, spectral unmixing, and data analysis

For further analysis, the MSOT images with slightest motion were selected utilizing the co-registered US images as reference. MSOT acquisitions at wavelength 1195 nm were discarded for all scans due to a very low signal-to-noise ratio triggered by strong absorption of the probe’s coupling pad. The remaining raw data of the selected frames was additionally pre-processed with a machine learning-based denoising algorithm [25]. This method strongly reduces the electrical noise present in the measurement data and thus, boosts morphological and spectral MSOT image quality, in particular the ability to access the rich multispectral contrast at high spatial resolution. Subsequently, the denoised signals were bandpass filtered (Butterworth filter, 0.5–12 MHz) before image formation. MSOT images were then reconstructed utilizing a custom model-based algorithm [26, 27]. The implementation is available in a public repository.^1^ The model-based image reconstruction strongly enhances image resolution and spectral contrast compared to standard back-projection methods in optoacoustics has been shown in previous publications [21, 26, 27, 31]. The algorithm performs a non-negativity constrained least squares minimization with an additional shearlet L^1^-regularization [32, 33]. The minimization problem was solved via bound-constrained sparse reconstruction by separable approximation. The algorithm requires 7 to 14 min to reconstruct an image with 14 wavelengths [34]. The speed of sound in the coupling medium of the probe and inside tissue applied for US propagation modelling were tuned manually for optimal image quality. In addition, the algorithm corrects for the total impulse response of the transducers, which has been shown to enhance the quality and fidelity of reconstructed images even further [21, 26]. The regularization parameter was optimized manually with an L-curve.

We employed linear spectral unmixing with literature spectra of HbR, HbO2, lipid, melanin, collagen and H2O on the complete MSOT dataset as performed in previous studies [17, 19, 20]. In addition, we performed blind spectral unmixing, which has shown to yield more distinct separation of molecular targets than linear unmixing, because it can account for distorting effects like spectral coloring [35]. For blind spectral unmixing, both the most significant (i.e., mathematically best matching) absorption spectra and their contributions to the intensity in each pixel (coefficients) are determined in a data-driven manner by performing a non-negative matrix factorization. L^2^-regularization was employed to decrease the effects of high-frequency noise, and L^1^-regularization ensured the biologically expected sparse contribution of absorbers to pixel intensities [36]. Both spectral unmixing algorithms were applied to scans of both thyroid nodules and healthy tissue of all 27 patients in post-processing. Linear unmixing required 7.35 s and blind unmixing 27.20 s per image. The number of blindly unmixed spectra was set to eight empirically and regularization parameters were optimized using an L-curve.

MS-OPUS scans of the four blind unmixing components with largest biological interpretability similar to HbO2, HbR, lipids, and H2O were used by two reviewers to evaluate all scans independently. This selection was performed by one medical doctor with research experience in nuclear, optical, and optoacoustic imaging of the head-and neck region and one engineer with research experience in optoacoustic imaging. The selected scans were discussed in the research team comprising mathematicians, radiologist, pathologist, and head neck surgeon. Scans which showed clinically relevant indicators of malignancy observable in MSOT were selected and compared with histopathology for the identification of possible malignancy markers.

Going beyond the analysis of single patients, we performed a statistical analysis to discriminate malignant from benign thyroid nodules in our complete data set. To reproduce the results of previous studies, we computed the mean coefficients of HbO2 and HbR over the thyroid nodule and the contralateral healthy thyroid utilizing the linear unmixing results. Oxygen saturation (SO2) was computed as ratio of HbO2 over total blood volume for each pixel [16–20]. Additionally, we also computed the mean coefficients of blindly unmixed spectra with largest biological interpretability linked to these chromophores. The thyroid nodule, contralateral healthy thyroid and other selected regions of interest (ROIs) were manually segmented in the US image and transferred to the co-registered MSOT image. Vessel diameters were determined as full-width-at-half-maximum (FWHM) along profile lines across the vessel. The profile lines were selected manually.

The mathematical equations for all algorithms applied for data processing, image reconstruction, spectral unmixing and data analysis are provided in the Supplementary Information. For all images, for which we computed mean spectra observed with MSOT and determined the Contrast Resolution (CR, see Supplementary Methods for definition) to quantify the contrast, we added the corresponding figures including ROI segmentations to Supplementary Fig. 7.

### Histopathological analysis

During surgery, the specimen was marked, and during the whole pathological process, the specimen’s orientation was documented. This made it possible to correlate the scans to histopathology and to check the availability of sufficient histopathological data to correlate MS-OPUS scans to histopathology. Because the MS-OPUS transducer’s sensitivity field leads to imaged cross-section of approximately 3-mm thickness, the histopathological slide (0.5–3um) with highest similarity to the MS-OPUS image inside this volume was selected. Histopathological data was discussed with a dedicated thyroid pathologist.

Immunohistochemical staining was performed at the Department of Pathology of the University Medical Center Groningen to assess microvascularity in malignant thyroid nodules and in normal thyroid tissue. Based on the location during the imaging of the patients, we selected tissue slides of malignant thyroid nodules and healthy thyroid tissue from the same cases. The selected slides were stained with anti-CD31 antibody, a vascular marker of angiogenesis, using a standardized protocol. Hematoxylin and Eosin (H&E) staining was already performed per standard protocol and available for correlation. Additionally, the microvessel density was scored by an expert pathologist as 0 (low) tot 5 + (very high) and correlated with the H&E slide and all vascular structures on the H&E pathology slides were marked. Vessel diameters were determined as the width of the manually segmented vessels on the H&E slides.

## Results

### Patient characteristics

In total, we asked 52 patients for consent (32 females and 20 males) of which 29 agreed to participate and met the inclusion criteria after screening. Thus, a total of 29 patients were enrolled, of which two patients were excluded due to technical failure of the MSOT device (one moment of technical failure of the laser trigger board in two patients scanned in succession), totaling 27 included patients in this study (Fig. 1). Fourteen patients were male (51.9%) and the median age was 60 years (IQR 51.0–66.0 years) (Table 1, Supplementary Table 2). All patients were Caucasian, making large differences in melanin content unlikely [37]. In total, we imaged 38 thyroid index nodules (Supplementary Table 3). A total of 17 patients underwent thyroidectomy leading to 27 index nodules with final histopathology data, totaling 11 malignant and 16 benign nodules. Cytology served as the gold standard in 10 patients, harboring four malignant nodules (Bethesda 6) and six benign nodules (Bethesda 2) (Supplementary Table 3). One patient with a Bethesda 4 nodule is still in follow-up for metastatic colorectal cancer, and due to the lack of final histopathology, was not included in the analysis.

**Table 1:** Patient baseline characteristics

Characteristics	Total (*n* = 27)
Sex, *n* (%)	
Male Female	14 (51.9%) 13 (48.1%)
Age (years), median (IQR)	60.0 (51.0 – 66.0)
TIRADS of index nodule, *n* (%)	
TIRADS 1 TIRADS 2 TIRADS 3 TIRADS 4 TIRADS 5 NA	1 (3.7%) 2 (7.4%) 6 (22.2%) 5 (18.5%) 7 (25.9%) 6 (22.2%)
Cytology of index nodule, *n* (%)	
Bethesda 1 Bethesda 2 Bethesda 3 Bethesda 4 Bethesda 5 Bethesda 6 NA	1 (3.7%) 7 (25.9%) 0 (0.0%) 5 (18.5%) 4 (14.8%) 8 (29.6%) 2 (7.4%)
Histopathology index nodule, *n* (%)	
Malignant (papillary thyroid cancer) Benign	10 (37.0%) 7 (25.9%)

*IQR*interquartile range, *NA* not available

### Spectral unmixing of MSOT images

**Fig. 2:**
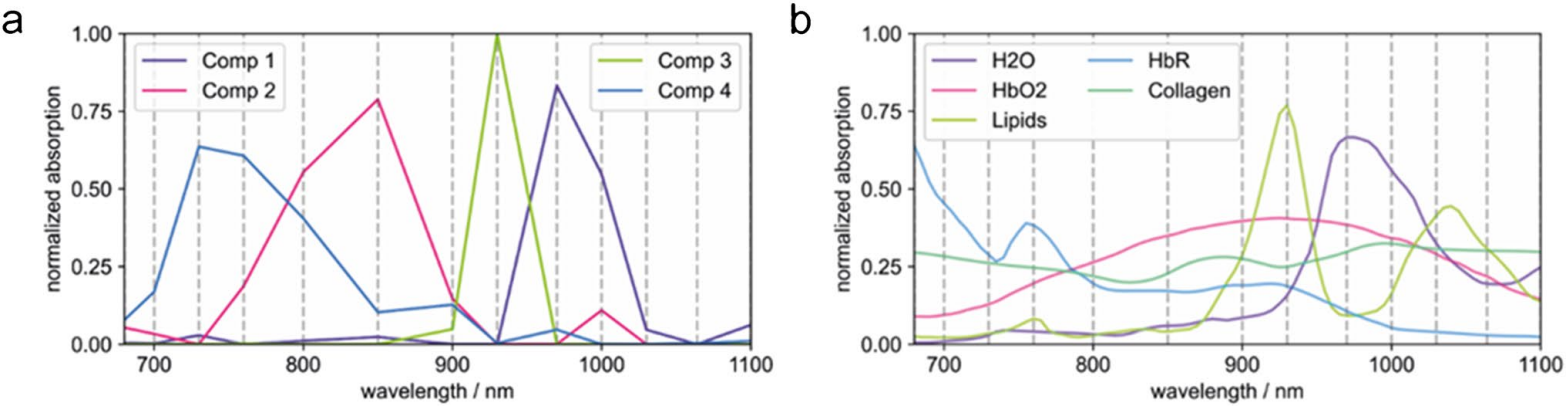
Absorption spectra of blindly unmixed components 1, 2, 3, and 4 (**a**) compared to reference absorption spectra of respective H2O, HbO2, lipid, HbR, and collagen (**b**). Colors and order of display are chosen to match similarities. Dashed lines represent the wavelengths at which MSOT images were acquired

Chromophores of interest for analyzing thyroid nodules were HbO2, HbR, lipids, H2O, and collagen. HbO2, HbR, and H2O were included, because a common feature in thyroid cancer is enhanced angiogenesis and abundance of microvascularity [38]. Lipids were included since several studies report significant alteration of lipid profiles in thyroid cancer compared with adjacent nontumor tissues or benign lesions [39, 40]. Also, collagen was analyzed since altered expression of collagen associated with tumorigenesis is reported in thyroid cancer [41]. Reference absorption spectra of chromophores of interest for analyzing thyroid nodules (HbO2, HbR, lipids, H2O, and collagen) and the blind spectral unmixing components with the strongest similarity are shown in Fig. 2. Supplementary Fig. 1 shows all blind spectral unmixing components. Throughout the manuscript we display blind unmixed MSOT images. For comparison we also included all linearly unmixed components for one scan in Supplementary Fig. 5. For blind unmixing, we could not identify a single component that linked solely to collagen. Melanin was included in linear unmixing, but was not investigated further, because it is not present in thyroid nodules. In addition, all patients were Caucasian with comparable skin color, so the optical absorption of melanin affecting the overall light penetration depth and spectral coloring did not have to be considered any further.

### Scan selection for detailed analysis of MS-OPUS images

In all 12 selected cases, MS-OPUS showed distinctive patterns. However, in 4 of 12 cases, MS-OPUS could not be correlated to histopathology as no histopathology data was available from the plane at which the MS-OPUS scan was performed. In the remaining eight cases, all MS-OPUS scans showed distinctive patterns on MS-OPUS that could be correlated to histopathology. The following sections will showcase five of these cases and correlate them with available histopathology. The remaining cases are displayed in Supplementary Fig. 6.

### Identification of thyroid vascularization on MS-OPUS validated by histopathology

**Fig. 3:**
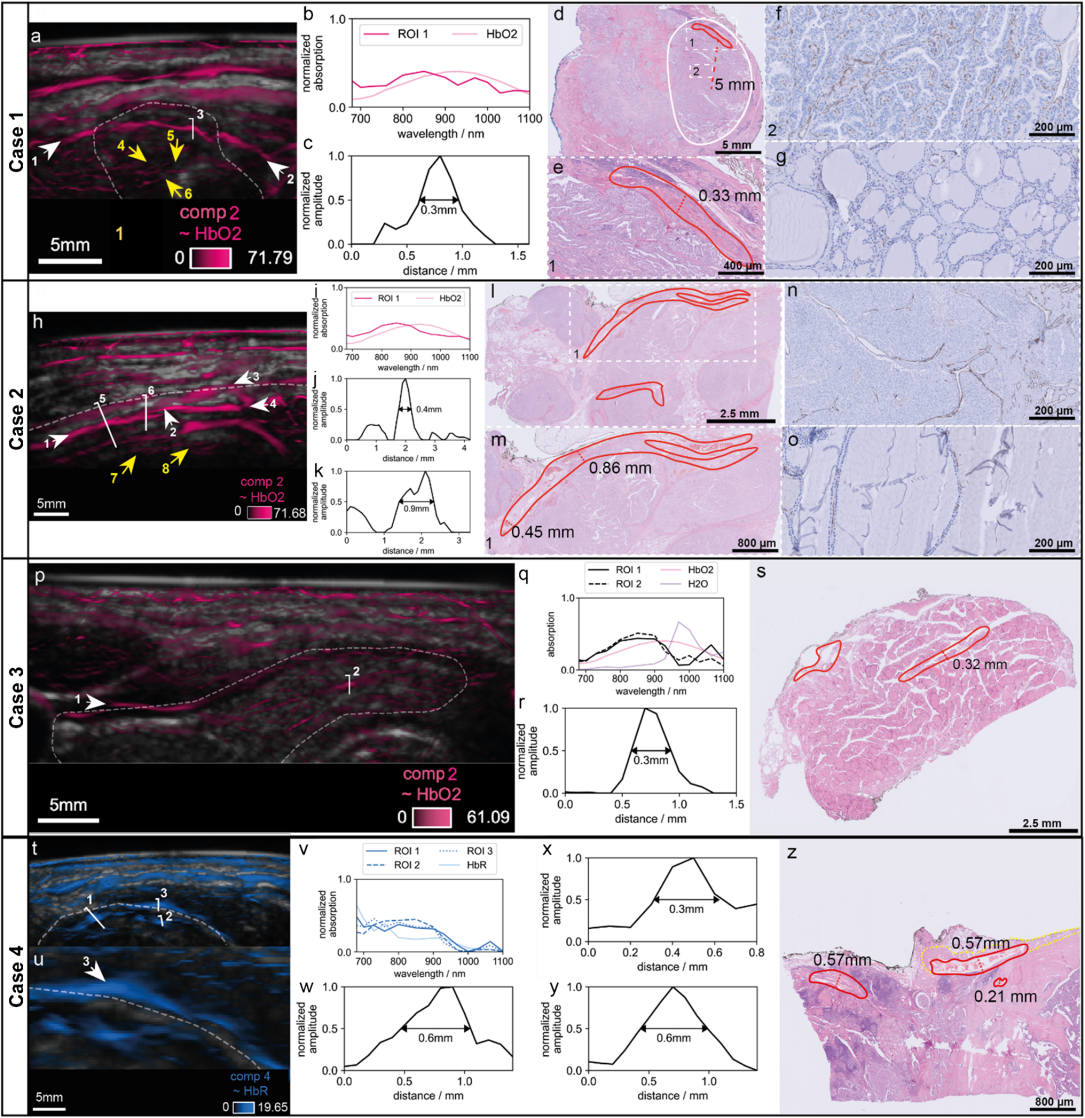
Four representative cases of spectral features in thyroid nodules with distinctive vascularization and high microvascularity in the nodule at high resolution correlated to histopathology. Case 1 (**a**–**g**), papillary thyroid carcinoma delineated in white. MS-OPUS (**a**) shows rich microvascularity (yellow arrows 4, 5, and 6, a more detailed image is added as Supplementary Fig. 4) in the thyroid nodule and a blood vessel (white arrows) crossing the cranial side of the nodule. The absorption spectrum is dominated by HbO2 (**b**, CR = 0.9999984). MSOT estimates a 0.3 mm vessel diameter along line 3 (**c**). Histopathology confirms the presence of this vessel with a diameter of 0.33 mm (**d**, **e**) and immunohistochemistry with anti-CD31 antibody shows higher microvascularity in the thyroid nodule (quantified as 5 + , **f**) than in the surrounding healthy thyroid tissue (quantified as 1 + , **g**). Case 2 (**h**–**o**), papillary thyroid carcinoma delineated in white. MS-OPUS (**h**) shows rich microvascularity in the thyroid nodule (yellow arrows 7 and 8, a more detailed image is added as Supplementary Fig. 4) and identifies a vessel with varying diameters which splits into two branches (white arrow 1 and splitting at white arrows 2 in 3 and 4). The absorption spectrum is dominated by HbO2 (**i**, CR = 0.9999601). MSOT estimates a vessel diameter ranging from 0.4 mm (line 5) (**j**) to 0.9 mm (line 6) (**k**). Histopathology confirms the presence of this vessel in the exact same shape and shows a diameter ranging from 0.45 to 0.86 mm (l and more detailed in **m**). Immunohistochemistry with anti-CD31 antibody showed higher microvascularity in the thyroid nodule (quantified as 3 + , **n**) compared to the contralateral healthy thyroid tissue (quantified as 1 + , **o**). Case 3, healthy thyroid tissue delineated in white. MS-OPUS shows two vessels, white arrow 1 and line 2 (P, arrow 1 CR = 0.9999853, line 2 CR = 0.9999928). The absorption spectrum is dominated by HbO2 (**q**) MSOT estimates a vessel diameter along line 2 of 0.3 mm (**r**). Both vessels are identified on histopathology, ROI 2 with a vessel diameter of 0.32 mm (**s**). Case 4, papillary thyroid cancer delineated in white with extrathyroidal extension. MS-OPUS (**t**, **u**) identifies three vessels (line 1 CR = 0.9999995, line 2 CR = 0.9999923 and line 3 CR = 0.9999950) with absorption spectrum similar to HbR (**v**). The vessel indicated by white arrow 3 (**u**), with its origin inside of the thyroid nodule but also entering the muscle on top of the nodule, visualizes extrathyroidal extension of the tumor in the surrounding muscles, as confirmed on histopathology (**z**, where red is vessels and yellow is muscle). MSOT estimates a vessel diameter along line 1 of 0.6 mm (**w**) along line 2 of 0.3 mm (**x**) and along line 3 of 0.6 mm (**y**), with matching diameters on histopathology (**z**)

We describe four cases (Case 1–Case 4) with distinctive findings in the MS-OPUS images which are substantiated by histopathology and could not be detected with US alone (see Supplementary Fig. 8 for pure US images of each Case). All patients were diagnosed with papillary thyroid cancer and underwent a total thyroidectomy. Figure 3 shows the findings per case with thyroid nodules delineated in white.

The first distinctive pattern visually identified on the MS-OPUS scans of all selected cases relates to vascular structures. Thyroid cancer commonly features enhanced angiogenesis and an abundance of microvascularity [38]. Moreover, aggregated vascular complexes have been identified in the stroma of tumor papillae, but not in the healthy thyroid or adenomas [38]. Therefore, vascularity may be a distinguishing feature for thyroid cancer. In order to investigate this more thoroughly, first the high-resolution visualization of vessels on MSOT must be validated. In order to do so, MS-OPUS blends (overlay of MSOT with co-registered US image) were correlated to final histopathology. In all four cases, the shapes and diameters of prominent vessels in the nodule on MSOT accurately reproduce histopathology sections (Fig. 3). For example, in Case 1, the MS-OPUS blend displays a blood vessel located 5 mm from the center of the nodule with a diameter up to 0.3, whose absorption spectrum is clearly dominated by HbO2 (Fig. 3, Case 1, panel A (white arrows), B (absorption spectrum), C (diameter of the vessel)). The presence of this vessel is histologically validated, showing a diameter of 0.33 mm and localization of 5 mm from the center of the nodule (Fig. 3, Case 1, panel D (vessel delineated in red), E (vessel delineated in red)). Case 2 also displays that shape and diameters measured at several cross-sections of the vessels on MS-OPUS (~ HbO2) closely resemble shape and diameters on corresponding histopathology sections (Fig. 3, Case 2,). In Case 3, we identify and confirm on histopathology two vessels in a healthy thyroid lobe on MS-OPUS again dominated by HbO2 absorption, including one vessel (ROI 1) deep in the tissue with a diameter of 0.3 mm and another one on the lateral cranial side of the thyroid gland (ROI 2) (Fig. 3, Case 3). The analysis of this patient’s papillary thyroid carcinoma is described as Supplementary Case 3 in Supplementary Fig. 2. In Case 4, we identify three vessels with strong HbR contrast at high resolution (Fig. 3, Case 4, panel T (white lines), U (white arrow), V (absorption spectrum). Because the absorption spectra of the selected ROIs show greatest similarity to HbR (see panel V), we display this component. The component similar to HbO2 is displayed in Supplementary Fig. 3. Two vessels are observed in the thyroid nodule and one in the surrounding muscle (ROI 3). We note that the vessel in ROI 3 has its origin inside of the thyroid nodule but also enters the muscle on top of the nodule, which could be explained by the presence of extrathyroidal extension in the surrounding muscles as determined with histopathology (Fig. 3, Case 4, panel Z (vessel delineated in red, muscle delineated in yellow). In fact, the diameter measured on MSOT at the cross-section of the vessel that invaded the muscle matches the diameter of the corresponding extrathyroidal vessel at histopathology (Fig. 3 Case 4, panel Y).

Collectively, the vessel diameters derived as FWHM on MS-OPUS showed an excellent correlation with the diameters of manually segmented vessels on histopathology (*n* = 7, 4 different cases, Fig. 3 panels B,C,I,J,K,Q,R,W,X,Y), with a regression analysis showing an R^2^-score of 0.9426.

### Microvascularity in thyroid nodules as potential malignancy marker identified on MS-OPUS

The second distinctive pattern we observe on MS-OPUS is the abundance of microvascularity in multiple malignant thyroid nodule cases, which could reflect tumor angiogenesis [38]. For example, in Case 1, component 2 shows very fine structures in high density up to a depth of 2 cm in addition to the previously described dominant vessel, which can be interpreted as high microvessel density (Fig. 3, Case 1, panel A, yellow arrow 4–6). Staining for anti-CD31 antibody, a vascular marker of angiogenesis shows high microvessel density throughout the complete nodule on the corresponding histopathology sections, which is also increased compared to surrounding healthy thyroid tissue (Fig. 3, Case 1, panels F and G). The same is observed in Case 2 (Fig. 3) and Case 3 (Supplementary Fig. 2).

### Non-significant differences between malignant and benign thyroid nodules by mean oxygen saturation and collagen architecture in the capsule

**Fig. 4:**
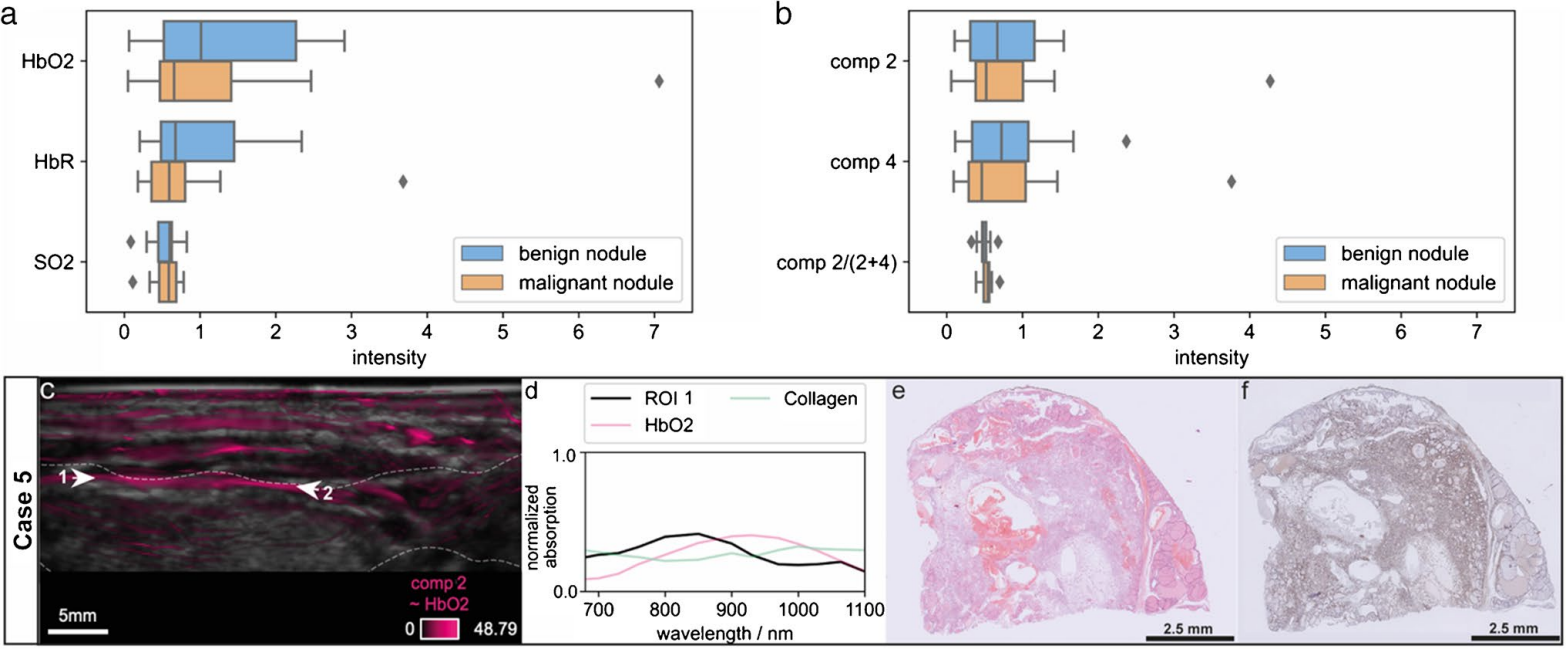
Box plots of the mean coefficients related to HbO2 and HbR, as well as SO2 in benign or malignant nodules obtained with spectral unmixing of the MSOT images and an exemplary case of a thyroid nodule with a prominent capsule of the thyroid gland. The mean coefficients for HbO2 and HbR over the nodule ROI and the corresponding SO2 obtained with linear unmixing (**a**) do not show any significant difference. Similarly, mean coefficients of components 2 (related to HbO2) and 4 (related to HbR) in the nodule ROI recovered from blind unmixing and their ratio (**b**) do not differ significantly. Case 5 (**c**–**f**) shows a patient with multinodular goiter.. The MS-OPUS blend displays strong MSOT contrast in the boundary region of the thyroid (Case 5, panel **c**, region between arrows 1 and 2, thyroid gland is delineated in white). The mean absorption spectrum of the thyroid capsule (between arrows 1 and 2, CR = 0.9999984) obtained from MSOT (Case 5, panel **d**) is very similar to HbO2 without specific features related to collagen. The corresponding tissue slide (**f**) shows collagen rich tissue below the capsule of the thyroid gland without any signal of collagen on MS-OPUS

Despite the high quality of the overall data set and the promising findings for single patients, we cannot reproduce significant differences between malignant from benign thyroid nodules in accordance to previous studies. Mean concentrations of HbO2 and HbR in the nodule ROI, as well as corresponding SO2 obtained with linear unmixing do not show a significant difference (Fig. 4, panel A). The same applies to the mean coefficients of component 2 (related to HbO2) and 4 (related to HbR) and their ratio, which are obtained by blind unmixing (Fig. 4, panel B). Thus, we fail to obtain quantitative results from our MSOT data with mean coefficients over the nodule ROI and are not able to determine a decrease in oxygen saturation like in previous studies [16–19].

Additionally, we are not able to identify collagen in thyroid nodules from MS-OPUS data. For example, Case 5 showcases a prominent capsule of the thyroid which is visible in the histology slide (Fig. 4, Case 5, panel E). Thyroid capsules are composed of connective tissue with significant collagen concentration [42]. MSOT also shows contrast in the boundary regions of the nodule (Fig. 4, Case 5, panel C, region between arrows 2 and 3). However, the mean absorption spectra of this ROI recovered from MSOT show greatest similarity to HbO2 (Fig. 4, Case 5, panel D). Thus, we are unable to detect the capsule on the MS-OPUS image, because strong perfusion is no unique capsule marker and presence of collagen cannot be recovered. Therefore, the proposed variations in collagen architecture of benign versus malignant nodules cannot be established.

Possible explanations, why we are neither able to establish a decrease in oxygen saturation for malignant thyroid nodules compared to benign ones nor quantify collagen in the thyroid nodule capsule from our MSOT data are discussed in the following paragraphs.

### Limitations of quantitative MSOT imaging

**Fig. 5:**
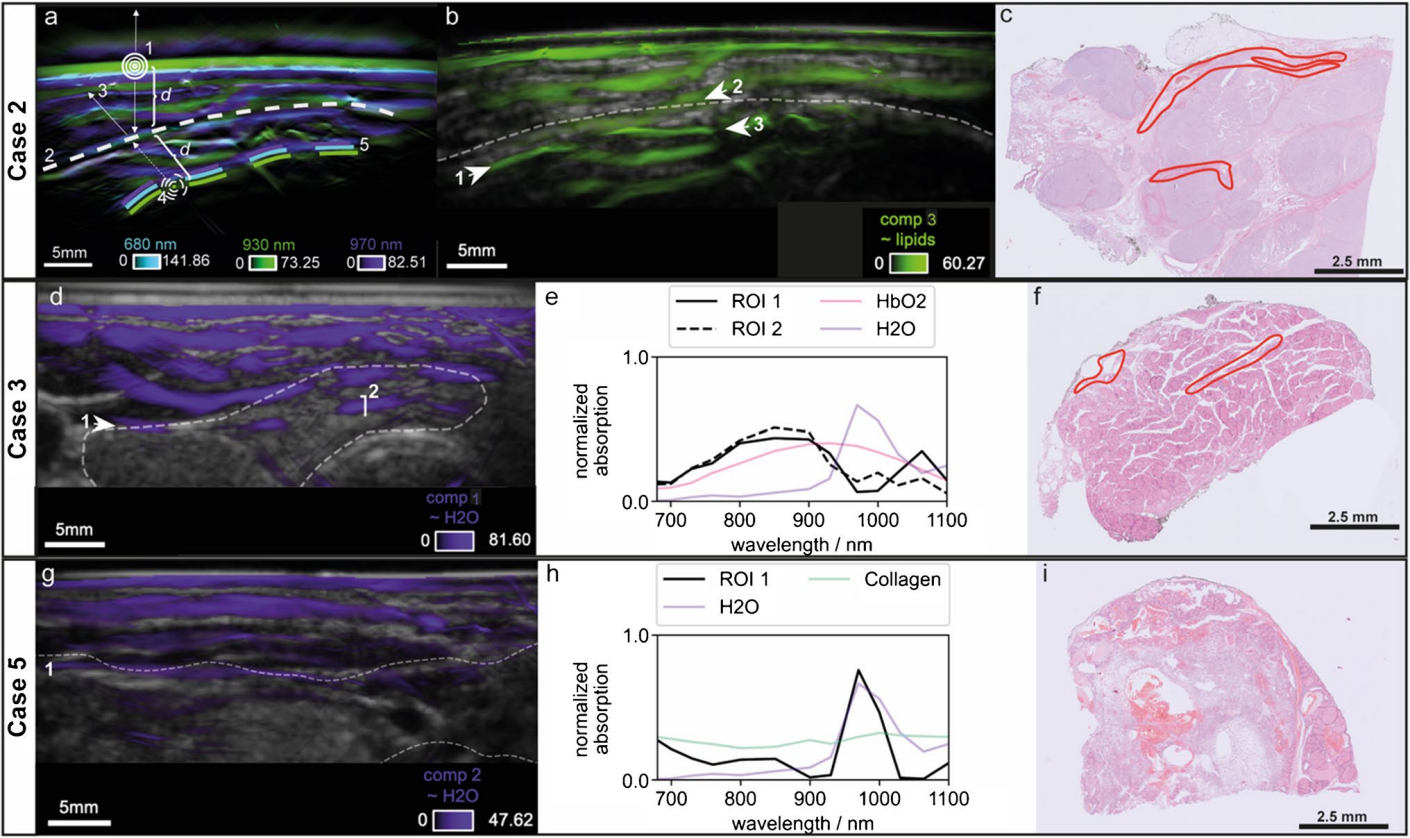
Reflection artifacts, spectral coloring, spectral cross talk, and masking by strong absorbers limiting quantitative MS-OPUS imaging. Case 2 (**a**–**c**), papillary thyroid carcinoma delineated in white. MSOT (**a**) exhibits reflection artifacts (dashed line 5) of the skin signal caused by the capsule of the thyroid nodule (dashed line 2). MSOT signals originating in point 1 are reflected at the capsule (arrow 3) and an artificial source (point 4) is mirrored to the opposite side. MS-OPUS (**b**) displays strong contrast for component 3 (similar to lipids) inside a vessel (between arrows 1, 2, and 3) which may be misinterpreted, as histopathology (**c**) verifies the presence of vessels in the thyroid nodule. In case 3 (**d**–**f**), a healthy thyroid gland delineated in white, MS-OPUS (D) unveils a notch in the spectrum of vessels (arrow 1 CR = 0.9999886, line 2 CR = 0.9999995) in the wavelengths range between 970 and 1000 nm (**e**) compared to the reference HbO2 absorption spectrum. This displays the effect of spectral coloring triggered by strong H2O absorption in the dermis region on top of the selected regions of interest. Histopathology (**f**) shows the presence of vessels in the thyroid nodule. Case 5 (**g**–**i**) shows a shows a patient with multinodulair goiter, thyroid gland delineated in white. MS-OPUS (**g**) exhibits reflection artifacts caused by multiple interfaces in the subcutaneous fat which mirror dermis contrast in the region of the muscle fascia (dashed line 1, CR = 0.9999994). The mean absorption spectrum along the muscle fascia (**h**) obtained from MSOT contains clear features of H2O absorption, but not of collagen which contradicts biology (**i**)

Major challenges for quantitative MSOT imaging arise from acoustic reflection artifacts [43]. Human tissue is naturally heterogeneous exhibiting multiple textures with varying acoustic properties (i.e., speed of sound and mechanical density). Thus, there is a mismatch in acoustic impedance between different textures which constitutes as a reflective interface for US waves. In MSOT, optical absorbers emit US waves in all spatial directions. Consequently, the wave of a single absorber may arrive at a US detector multiple times. The first incidence corresponds to the direct impacting wave, whereas any following can be regarded as reflection. If the physical optoacoustic model only accounts for direct wave propagation, the signal origin of the reflected wave will not be localized correctly. An artificial optoacoustic source mirrored to the opposite side of the reflective interface will emerge in the image (Fig. 5, Case 2, panel A, point 4 with equal distance *d* to line 2 as point 1). Thus, a reflection of the MSOT skin signal can be observed inside the nodule in Case 2 (Fig. 5, Case 2, panel A, line 5). Reflections of the optoacoustic signal of the membrane may be misinterpreted as lipid abundance inside the thyroid nodule, contradicting the findings from histopathology that identify no lipids in this region (Fig. 5, Case 2, panel C). Especially if reflection artifacts superimpose optoacoustic signals of other tissue like in Case 5, they hamper quantitative MSOT imaging. Although the ROI along line 1 (Fig. 5, Case 5, panel G) can clearly be identified as muscle fascia in the US image, the H2O-dominant absorption spectrum provided by MSOT (Fig. 5, Case 5, panel H) does not match with the histological composition of muscle fascia (i.e., strong blood perfusion and collagen, Fig. 5, Case 5, panel I). A thorough investigation of the anatomy in the US image shows multiple interfaces in the subcutaneous fat which mirror strong H2O contrast of the dermis in the ROI of the muscle fascia. We would like to emphasize that vascular patterns of malignancy identified in single cases in this study (see paragraphs 3.4 and 3.5) were ruled out to be reflection artifacts by five experts.

Quantitative MSOT imaging is also impeded by limited penetration depth due to strong optical absorbers like hemoglobin and melanin in superficial skin layers. Light attenuation strongly varies significantly depending on the positioning of the probe on the skin, the skin tone [37] and the heterogeneous tissue. The stronger decay of light fluence limits the imaging depth, because the optoacoustic signal generated by absorbers is decreasing more rapidly, resulting in a lower signal-to-noise ratio [44]. Moreover, the heterogeneous tissue introduces the effect of spectral coloring, which refers to the phenomenon of distorted optical absorption spectra recovered from MSOT data compared to references from literature. This variation is caused by inadequate modeling of light fluence decay inside tissue for different wavelengths [36]. Mean absorption spectra in ROIs 1 and 2 in Case 3 display strong similarity to the absorption of HbO2 (Fig. 5, Case 3, panel E). This is biologically reasonable, because histopathology unveils a blood vessel in the healthy thyroid in this region (Fig. 5, Case 3, panel F). However, the mean absorption spectra determined with MSOT denote a clear notch in the wavelength range between 970 and 1000 nm compared to the reference HbO2 absorption spectrum. This notch displays the effect of spectral coloring and is triggered by strong H2O absorption in the dermis region on top of the selected ROIs. Because H2O strongly absorbs light at the wavelength range between 970 and 1000 nm (Fig. 5, Case 3, panel E), fluence decreases faster with depth for these wavelengths than for other wavelengths. However, this variation in fluence decay for different wavelengths is not incorporated in the physical model used for image reconstruction. Light absorption deep in tissue for the two wavelengths (970 and 1000 nm) will thus appear to be decreased resulting in distorted absorption spectra. Inherently, the quantification of biomarker presence utilizing linear unmixing with reference literature absorption spectra will be affected negatively.

In our data set, we observed a relative mean squared error of 9.03% for linear unmixing. In other words, 9.03% of the MSOT image contrast cannot be explained with the contribution of the biomarkers selected for unmixing. Because blind spectral unmixing recovers the mathematically best matching absorption spectra from the measurement data itself, it can account for the distortions caused by spectral coloring. Therefore, blind spectral unmixing generally provides a more authentic representation of MSOT measurement data than linear unmixing resulting in a relative mean squared error of only 0.84% in this data set [35]. Yet, interpretation of blind unmixing results remains challenging, because only some of the recovered absorption spectra can be linked to specific chromophores (Fig. 1).

In addition to reflection artifacts, reduced depth penetration and spectral coloring, scientists may erroneously interpret optical absorption as a mixture of different chromophores. This phenomenon of spectral cross talk also deteriorates image quantification [45]. For example, a large artery can clearly be identified in Case 2 (Fig. 3, Case 2, panel H, region between arrows 1, 2, 3 and 4) by the mean optical absorption spectrum in the MSOT image (Fig. 3, Case 2, panel I). The corresponding histology section confirms this observation displaying a vessel of equal size and shape (Fig. 3, Case 2, panel L). As expected, blind spectral unmixing will recover strong contributions by a component with absorption similar to HbO2 (component 2) in this ROI (Fig. 3, Case 2, panel I). However, the absorption spectrum of component 2 features weaker absorption at wavelengths above 850 nm compared to the literature absorption of HbO2 (Fig. 2, panels A and B). In order to account for this deviation, HbO2 contrast will resemble a mixture of components by blind unmixing. Consequently, also component 3 exhibits strong intensity in ROI 1 in Case 2 to account for the absorption at 930 nm (Fig. 5, Case 2, panel B). If component 3 was to be utilized independently for biomarker quantification due to its strong similarity to the optical absorption of lipids, presence of lipids surrounding the artery could be erroneously concluded by the scientist. However, this interpretation contradicts findings in the corresponding histology slide (Fig. 5, Case 2, panel C).

Ultimately, chromophore quantification from MSOT may also be distorted because strong optical absorbers cover weak ones in MSOT [46]. For example, tissue with high collagen concentration, like connective tissue (including collagen) in the capsule of the thyroid gland and thyroid nodule, is very often also strongly perfused [42]. HbO2 and HbR trigger stronger signal in the acquired near-infrared wavelength range compared to other chromophores like collagen [47]. Additionally, the absorption spectrum of collagen does not contain any isolating features in this optical range which would enable a differentiation from other absorbers [29]. We hypothesize a cover up by stronger absorbers is the case in Case 5 of this study (Fig. 5, panel c–f). The patient was diagnosed with multinodular goiter. The immunohistochemistry image shows a collagen rich thyroid gland (Fig. 5, panel f). However, MSOT only shows HbO2 and HbR related contrast located in the thyroid capsule (Fig. 5, panel c, between arrow 1 and arrow 2) without any signal attributable to collagen The absence of collagen signal in MSOT may be a result of a cover up by strong optical absorbers like HbO2 and HbR (refer to 3.6 and Supplementary Fig. 5 for linear unmixing images).

## Discussion

In this pilot study, we apply previously enhanced signal processing and image reconstruction methods for clinical MS-OPUS to provide unprecedented image quality in optoacoustic imaging of thyroid nodules. These features could, for the first time, be linked directly to reference histopathology data. In correlation with histopathology, we identify possible features of malignancy visible in MS-OPUS, such as abundance of microvascularity in malignant thyroid nodules, and visualize extrathyroidal extension in the surrounding muscle. We showcase representative examples seen in five cases. The techniques and findings presented herein provide, to the best of our knowledge, the highest quality optoacoustic images of thyroid nodules to date.

Our approach shows the ability to resolve blood vessels with diameters as small as 250 μm at depths of up to 2 cm. We surmise that the microvessel density in deeper levels of the nodules could not be visualized with MSOT due to decreasing light fluence. To validate our results, we linked the MS-OPUS scans directly to histopathology data, showing an excellent correlation between the vessel diameter measured on MS-OPUS and histopathology. Comparable imaging quality for thyroid nodules has so far not been reported. Despite the lower resolution of MSOT compared to microscopy applied in histopathology and an estimated 10% shrinkage of the tissue during the fixation process [48, 49], we expect similar vessel diameters in both modalities. The sparsity regularization in the Shearlet domain enhances edges like small vessels [50]. Because we reconstructed images at 100 µm resolution, vessels can be reconstructed with higher precision than the physical resolution would suggest. Moreover, diameters in MSOT were determined as FWHM along a profile line, whereas the diameters in histopathology were determined as widths of the manually segmented vessel in the image. The validated MS-OPUS scans were subsequently used to study vascular patterns as a distinguishing feature for thyroid cancer, as it is known that thyroid cancer, on pathology level, shows enhanced angiogenesis, distinctive morphological features and abundance of microvascularity [38]. We found that MSOT may be capable of identifying an abundance of microvascularity in malignant thyroid nodules. Previous publications demonstrated the ability of MSOT to image vessels with resolution up to 200 μm at depths up to 2 cm in varying tissue types [21, 26, 51–54]. Therefore, MS-OPUS can potentially improve detection of malignant thyroid nodules preoperatively based on the microvessel density. Furthermore, as a result of the validated MS-OPUS scans, we are able to show the presence of extrathyroidal extension of the nodule into a surrounding muscle. Literature shows that the extent of extrathyroidal extension is an adverse prognostic factor in thyroid carcinomas associated with higher recurrence risk and lower survival [55, 56]. In fact, in the American Thyroid Association guidelines of 2015, the presence of extrathyroidal extension is one of the features that implies high malignancy risk (> 70–90%) [7]. The presence of extrathyroidal extension in a differentiated thyroid carcinoma will place the patient into a high-risk group, which mandates a more aggressive treatment strategy consisting of total thyroidectomy and post-operative radioactive iodine therapy [7, 57, 58]. This treatment strategy has a major impact on patients caused by hypothyroidism resulting in the need of thyroid hormone replacement therapy and thereby affecting the quality of life. Total thyroidectomy is associated with complications such as iatrogenic hypoparathyroidism, dysgeusia and xerostomia also resulting in a poor quality of life [59–61]. The sensitivity and specificity of US in detecting extrathyroidal extension varies from 62.9–65.2% to 81.8–97.6% [62]. Here, we show the potential of MSOT to serve as a non-invasive technique to qualitatively assess the presence of malignancy and extrathyroidal extension, allowing for a more aggressive and thus appropriate treatment (i.e., surgery and dose of radioactive iodine).

Earlier studies on MSOT imaging of the thyroid suggest its potential to differentiate malignant from benign nodules and normal human thyroid tissue based on differences in mean HbR, HbO2, and SO2 concentrations in the nodule region [16–20]. Furthermore, the architecture of collagen varies in benign versus malignant thyroid nodules capsules and collagen could help detect the disruption of the capsule or the thyroid gland, which is a sonographic finding associated with extrathyroidal extension [42, 63]. Consequently, MS-OPUS might provide information about an additional malignancy marker in vivo. Although in the current study, we achieve the highest quality, i.e., spatial resolution and spectral contrast optoacoustic images of thyroid nodules to date, we are unable to replicate the results of these previous MSOT studies. Thus, we were not able to find statistically significant differences between malignant and benign thyroid nodules based mean coefficients over the thyroid ROI. To further investigate, we present general limitations of the field of optoacoustics inhibiting quantification of biomarker presence. Image artifacts due to acoustic reflections cause non-reasonable contrast superimposing optoacoustic signals in the same ROI. Spectral coloring due to unaccounted light fluence variation distorts the absorption spectra of chromophores recovered from MSOT images. Furthermore, strong optical absorbers both limit the light penetration depth due to their strong presence in superficial skin layers and can mask optical absorption of other chromophores like collagen. Finally, for the acquired wavelengths, the absorption spectrum of collagen does not exhibit unambiguous features like highest optical absorption or individual variations.

These limitations combined strongly impede the quantification of chromophore presence from MSOT. In addition, the mean is a measure of the absorbed energy in the region and thus, strongly dependent on nodule size, the segmentation accuracy, and the overlaying tissue with varying light absorption. Consequently, we emphasize that visual inspection of standalone MSOT images can lead to wrong interpretation. Inferring the presence of intrinsic biomarkers based on the mean absorption spectrum in a defined ROI applying spectral unmixing techniques might contradict biological reality. Therefore, we recommend to also look for qualitative optoacoustic features of malignancy within a ROI rather than only quantifying the mean absorption spectrum within a ROI.

Our small cohort limits the utility of establishing patterns predictive of malignancy in thyroid nodules. A future validation study with a more extensive data set should compare histopathology and MS-OPUS images for more patients and confirm our findings. Moreover, it may succeed using the applied data processing and image reconstruction methodology. The present study was the first step in identifying such possible optoacoustic features that could be used for the characterization of thyroid nodules.

In this study, by coincidence only papillary thyroid cancer or benign thyroid nodules were included. In a subsequent study, different types of thyroid cancers (such as follicular thyroid cancer) and benign lesions should be included to study if our results can be translated to other types of thyroid nodules. Future studies should also focus on diminishing artifacts in MSOT images, since artifacts may lead to wrong interpretation of images, possibly resulting in inadequate diagnosis and treatment in clinical applications. This requires training of clinicians to decrease the number of artifacts resulting from data acquisition. Algorithmic improvements incorporating optical tissue priors and acoustic ultrasound information as well as uncertainty quantification will both mitigate artifacts like acoustic reflections and enhance the data analysis facilitating the interpretation [64–66]. Additionally, MSOT probes could be optimized for deep tissue penetration and MSOT images should be acquired at more wavelengths in the selected wavelength range. This may lead to more distinct spectral unmixing results enabling a clearer detection of HbO2 and HbR absorption and the differentiation of different collagens as potential markers of malignant thyroid nodules [67]. For an analysis of a large MS-OPUS data set, we advocate to extend the statistical analysis beyond mean and standard deviations in selected ROIs. Metrics like spectral or spatial correlations of optical absorbers should be investigated to substantiate proposed findings and quantifications [68]. The application of novel machine learning methods could enhance image quality further and unveil more complex optoacoustic features. Ultimately, it may improve the understanding of MSOT contrast beyond the state-of-the-art and help to predict malignancy in thyroid nodules. At the current stage, a trained operator is still necessary to analyze MS-OPUS images. We assume that MS-OPUS will contribute to the diagnostic process of thyroid nodules in the future. Thereby, overtreatment (i.e., diagnostic hemithyroidectomy) of patients with thyroid nodules and the inherent postoperative morbidity could be decreased massively leading to a higher quality of life for patients with thyroid nodules.

## Supplementary Information

Below is the link to the electronic supplementary material.

Supplementary file1 (DOCX 13.0 MB)

## Data Availability

The datasets generated during and/or analyzed during the current study are available from the corresponding author on reasonable request.

## Footnotes

1 https://github.com/juestellab/mb-rec-msot.

## Abbreviations

BSRTC: Bethesda System for Reporting on Thyroid Cytopathology
FNA: Fine-needle aspiration
HbO2: Oxyhemoglobin
HbR: Deoxyhemoglobin
H&E: Hematoxylin & eosin
H2O: Water
MSOT: Multispectral optoacoustic tomography
MS-OPUS: Ultrasound-multispectral optoacoustic tomography
ROI: Region of interest
SO2: Oxygen saturation
US: Ultrasound
