# Supplementary material for: Towards in vivo characterization of thyroid nodules suspicious for malignancy using multispectral optoacoustic tomography
Source: Eur J Nucl Med Mol Imaging. 2023 Apr 11;50(9):2736–50. doi: 10.1007/s00259-023-06189-1 (PMC10317911; doi:10.1007/s00259-023-06189-1)
Supplement: Supplementary file 1 — Supplementary file1 (DOCX 13.0 MB) [file 259_2023_6189_MOESM1_ESM.docx]

**Towards in vivo characterization of thyroid nodules suspicious for malignancy using multispectral optoacoustic tomography**

*European Journal of Nuclear Medicine and Molecular Imaging*

Milou E Noltes, Maximilian Bader, Madelon JH Metman, Jasper Vonk, Pieter J Steinkamp, Jan Kukačka, Henriette E Westerlaan, Rudi AJO Dierckx, Bettien M van Hemel, Adrienne H Brouwers, Gooitzen M van Dam, Dominik Jüstel, Vasilis Ntziachristos, Schelto Kruijff

*Corresponding author*

Prof. Dr. Schelto Kruijff, MD PhD

University Medical Center Groningen

Department of Surgical Oncology

Email: [s.kruijff@umcg.nl](mailto:s.kruijff@umcg.nl)

**Supplementary Materials**

**Methods**

*Data processing*

Detailed equations of applied methods:

Model-based image reconstruction[26,27]:

$$p_{0}^{*}=argmin_{p_{0}\geq0}\left\| Mp_{0}-s \right\|_{2}^{2}+\lambda_{\Phi}\Phi(p_{0})$$

$p_{0}$ (dimension: $n_{pixels}\times1$, $n_{pixels}$: total number of pixels in single image) denotes as the initial pressure distribution, which will be visualized in the MSOT image. $M$ represents the model matrix including the physics of imaging (i.e., acoustic wave propagation and transducer characteristics, dimensions: $\left( n_{transducers}\cdot n_{time} \right)\times n_{pixels}$, $n_{transducers}$: number of acoustic transducers in array, $n_{time}$: number of time samples recorded by single transducer). The product $Mp_{0}$ provides the simulated signals for the proposed initial pressure distribution $p_{0}$. $s$ is the set of signals recorded during measurements (dimensions: $(n_{transducers}\cdot n_{time})\times1$). $\Phi$ represents the shearlet L1-regularization function [27,28] and $\lambda_{\Phi}$ the corresponding regularization parameter. The algorithm perfoms a regularized least-squares minimization with non-negativity constraint.

Linear Unmixing:

$$W^{*}=argmin_{W\geq0}\frac{1}{2}\left\| WH-S \right\|_{2}^{2}+\frac{\lambda_{W,2}}{2}\left\| W \right\|_{2}^{2}+\lambda_{W,1}\left\| W \right\|_{1}$$

$W$ denotes as the unmixing coefficients (dimension: $(n_{images}\cdot n_{pixels})\times n_{absorbers}$, $n_{images}$: total number of images in data set, $n_{absorbers}$: number of absorbers assumed to be present in tissue), $H$ are the absorption spectra of the absorbers sampled at the imaged wavelength (dimension: $n_{absorbers}\times n_{wavelength}$, $n_{wavelength}$: Number of wavelength acquired for an image) and $S$are the image pixels (dimension: $(n_{images}\cdot n_{pixels})\times n_{wavelength}$) and the regularization parameters $\lambda_{W,2}$, $\lambda_{W,1}$. In order to determine the unmixing coefficients $W$, this linear least-squares problem with regularization is minimized.

Blind Unmixing:

$$W^{*},H^{*}=argmin_{W,H\geq0}\frac{1}{2}\left\| WH-S \right\|_{2}^{2}+\frac{\lambda_{W,2}}{2}\left\| W \right\|_{2}^{2}+\frac{\lambda_{H,2}}{2}\left\| H \right\|_{2}^{2}+\lambda_{W,1}\left\| W \right\|_{1}+\lambda_{H,1}\left\| H \right\|_{1}$$

Blind unmixing defers from linear unmixing as it determines both the unmixing coefficients W and the most prominent absorbers with their spectra H. Both an L1- and L2-regularization for both coefficients and absorption spectra is applied with the corresponding parameters $\lambda_{W,2}$,$\lambda_{H,2}$,$\lambda_{W,1}$,$\lambda_{H,1}$. This minimization problem constitutes a non-negative matrix factorization with regularization.

Oxygen saturation:

$$SO_{2}=\frac{HbO_{2}}{HbR+HbO_{2}}$$

Oxygen saturation can be determined as the ratio of oxyhemoglobin ($HbO_{2}$) over total blood volume ($HbR+HbO_{2}$, $HbR$: deoxyhemoglobin).

Regression analysis:

$$R^{2}\left( w_{hist},w_{MSOT} \right)=1-\frac{\sum_{i=1}^{N} \left( w_{hist,i}-w_{MSOT,i} \right)^{2}}{\sum_{i=1}^{N} \left( w_{hist,i}-\overline{w}_{hist} \right)^{2}}$$

$w_{hist,i}$ denotes as width of vessel i determined in histopathology, $w_{MSOT,i}$ as width of vessel I determined from MSOT and $\overline{w}_{hist}$ as the mean of vessel widths determined from histopathology ($\overline{w}_{hist}=\frac{1}{N}\sum_{i=1}^{N} w_{hist,i}$). $N$ is the total number of vessels investigated.

Image and Unmixing accuracy metrics - normalized Mean-squared-error (MSE) [59]:

- Reconstruction algorithm: $MSE_{norm}=\frac{\left\| Mp_{0}-s \right\|_{2}^{2}}{\left\| s \right\|_{2}^{2}}$
- Linear/Blind Unmixing: $MSE_{norm}=\frac{\left\| WH-S \right\|_{2}^{2}}{\left\| S \right\|_{2}^{2}}$

The MSE determines, how accurate the reconstruction and unmixing algorithm approximates the given data (reconstruction: signal data $s$, spectral unmixing: image pixels $S$). More specifically, it is the squared residual of the minimization problem solved excluding regularization factors. Figuratively speaking both methods compare a simulation of their prediction (reconstruction: initial pressure $p_{0}$, linear unmixing: coefficients $W$, blind unmixing: coefficients $W$ and absorber spectra $H$) with the given data. Consequently, the lower this error is, the better the algorithms approximate the data. Any remaining error results from inaccuracies in the model (reconstruction: model matrix $M$, linear unmixing: absorber spectra $H$), e.g. unaccounted electrical noise of detection electronics in the reconstruction model or spectral coloring in linear unmixing. To give this metric independent of the energy of the given data which facilitates comparability, we normalize by the squared sum of the given data.

Contrast Resolution (CR) [25]:

$$CR=\frac{s_{mean,ROI}^{2}-s_{background}^{2}}{s_{mean,ROI}^{2}+s_{background}^{2}}$$

The CR provides a different contrast metric compared to the pSNR. It is calculated as the difference between mean intensity in the region of interest ($s_{mean,ROI}^{2}$) and the mean intensity of the background ($s_{background}^{2}$). The result is normalized by the sum of both mean intensities to provide a value in the range between zero and one. The region of interest and the background region are both segmented manually.

**Supplementary Tables**

**Supplementary Table 1. The Besthesda system for reporting thyroid cytopathology: Diagnostic categories and risk of malignancy**[10, 11]

| **Diagnostic category** | **Risk of malignancy^1^** |
| --- | --- |
| 1 – Nondiagnostic or Unsatisfactory  2 – Benign  3 – Atypia of Undetermined Significance or Follicular Lesion of Undetermined Significance  4 – Follicular Neoplasm or Suspicious for a Follicular Neoplasm  5 – Suspicious for Malignancy  6 – Malignant | 5 – 10%  0 – 3%  10 – 30%  25 – 40%  50 – 75%  97 – 99% |

| **Supplementary Table 2. Detailed patient characteristics.** | | | | | | | |
| --- | --- | --- | --- | --- | --- | --- | --- |
| **Patient number** | **Age in years** | **Gender** | **TIRADS**  **classification index nodule** | **Cytology, Bethesda classification index nodule** | **Histopathology,**  **index nodule** | **Diameter of index nodule on histopathology in mm** | **Selected for scan analysis** |
| 3 | 74 | Male | 5 | 6 | N/A | N/A | No |
| 4 | 70 | Female | 4 | 2 | N/A | N/A | No |
| 5 | 85 | Male | N/A | 6 | N/A | N/A | No |
| 6 | 37 | Female | 3 | 4 | PTC | 25 | No |
| 7 | 40 | Female | 5 | 6 | PTC | 17 | Yes, Case 1 |
| 8 | 20 | Male | N/A | 5 | PTC | 32 | No |
| 9 | 62 | Female | 3 | 4 | Benign (hurthle cell neoplasm) | 35 | No |
| 10 | 68 | Male | N/A | N/A | PTC | 95 | Yes, Case 2 |
| 11 | 50 | Female | 3 | 6 | PTC | 30 | No |
| 12 | 53 | Male | 5 | 4 | N/A | N/A | No |
| 13 | 50 | Female | 3 | 4 | Benign (hurthle cell neoplasm) | 28 | Yes |
| 14 | 63 | Female | 3 | 2 | N/A | N/A | No |
| 15 | 52 | Male | 5 | 6 | N/A | N/A | No |
| 16 | 47 | Female | N/A | 2 | Benign (follicular adenoma) | 55 | Yes |
| 17 | 61 | Male | N/A | N/A | Benign (hyperplastic nodule) | 20 | Yes |
| 18 | 59 | Male | 5 | 5 | PTC | 9 | Yes |
| 19 | 61 | Male | N/A | 6 | PTC | 80 | No |
| 20 | 61 | Male | 2 | 2 | N/A | N/A | No |
| 21 | 66 | Female | 4 | 6 | N/A | N/A | No |
| 22 | 43 | Female | 5 | 6 | PTC | 23 | No |
| 23 | 65 | Female | 3 | 4 | Benign (thyroid adenoma) | 90 | Yes |
| 24 | 58 | Female | 5 | 2 | Benign (colloid nodule) | 52 | Yes, Case 5 |
| 25 | 61 | Male | 4 | 2 | N/A | N/A | No |
| 26 | 20 | Male | 4 | 5 | PTC | 30 | Yes, Case 3  (nodule + healthy lobe) |
| 27 | 67 | Female | 4 | 2 | N/A | N/A | No |
| 28 | 54 | Male | 5 | 5 | PTC | 45 | Yes, Case 4 |
| 29 | 52 | Female | 3 | 1 | Benign (nodular hyperplasia) | 16 | No |
| N/A= not available; PTC= papillary thyroid carcinoma. | | | | | | | |

| **Supplementary Table 3.** Final classification of thyroid nodules. |
| --- |

| **Characteristics** | **Total** (n=38) |
| --- | --- |

| **Final classification nodule based on cytology,** n (%) | 11 (28.9%) |
| --- | --- |

| Bethesda 2  Bethesda 4  Bethesda 6 | 6 (15.8%)  1 (2.6%)  4 (10.5%) |
| --- | --- |
| **Final classification nodule based on histopathology,** n (%)  Malignant (papillary thyroid cancer)  Benign | 27 (71.1%)    11 (28.9%)  16 (42.1%) |

|  |
| --- |

**Supplementary Figures**


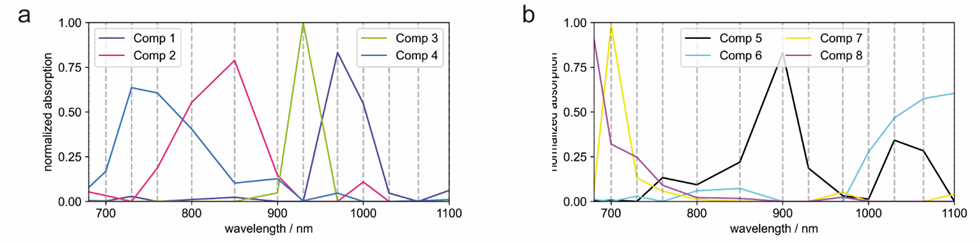
 **Supplementary Figure 1. Spectra of components 1, 2, 3 and 4 (a) and 5, 6, 7, and 8 (b) obtained with blind spectral unmixing.** Mathematically, the data set can be separated into contributions by these three components with the lowest unmixing error. Dashed lines represent the wavelengths at which MSOT images were acquired.


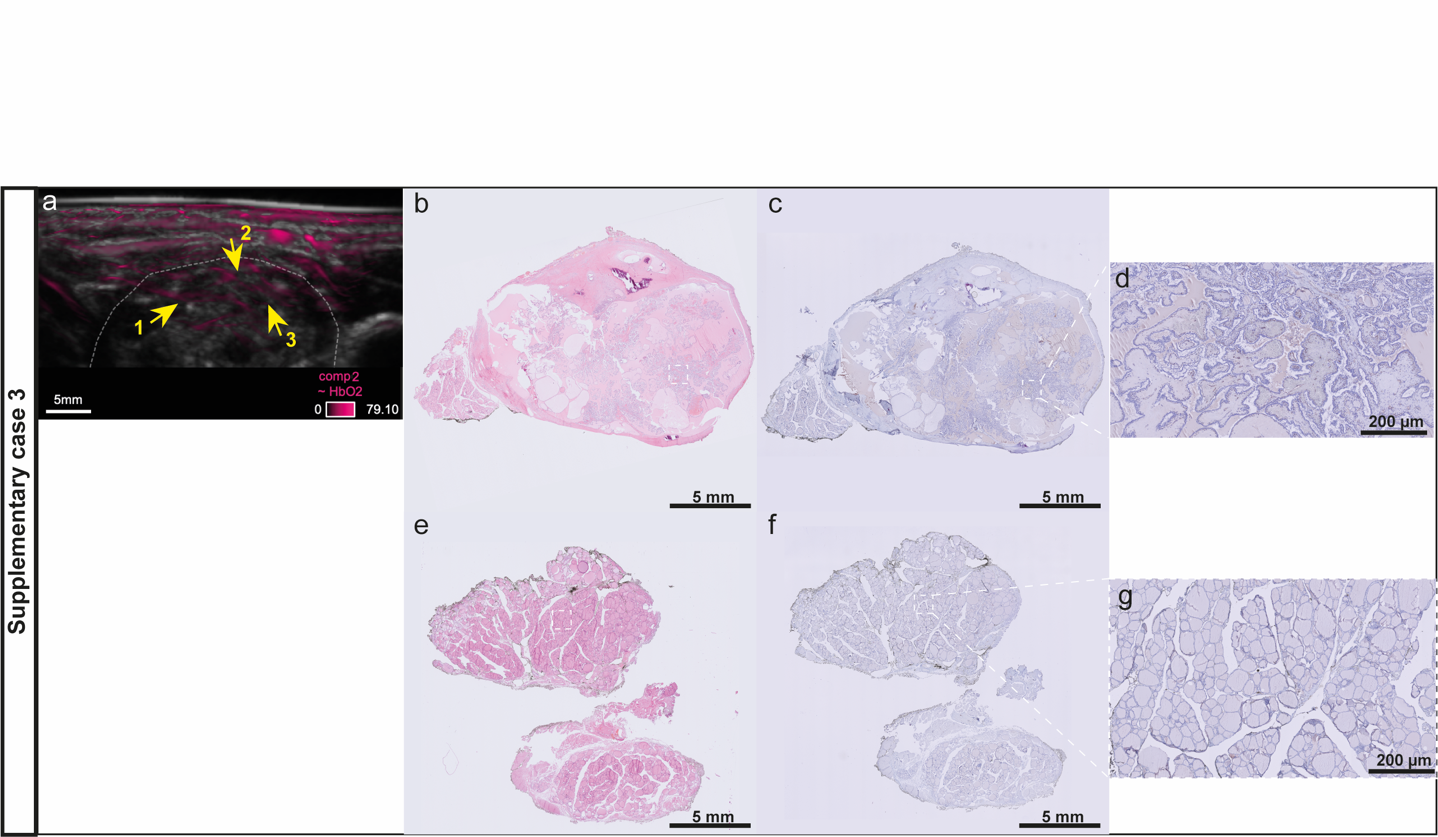


**Supplementary Figure 2. Papillary thyroid carcinoma of Case 3.** MS-OPUS (a) shows rich microvascularity in the thyroid nodule (yellow arrows). Immunohistochemistry with anti-CD31 antibody shows higher microvascularity in the malignant thyroid nodule (c, d, quantified as 3+) than in the contralateral healthy thyroid tissue (f, g quantified as 1+). Hematoxylin and Eosin (b, e) stained tissue sections are added for correlation


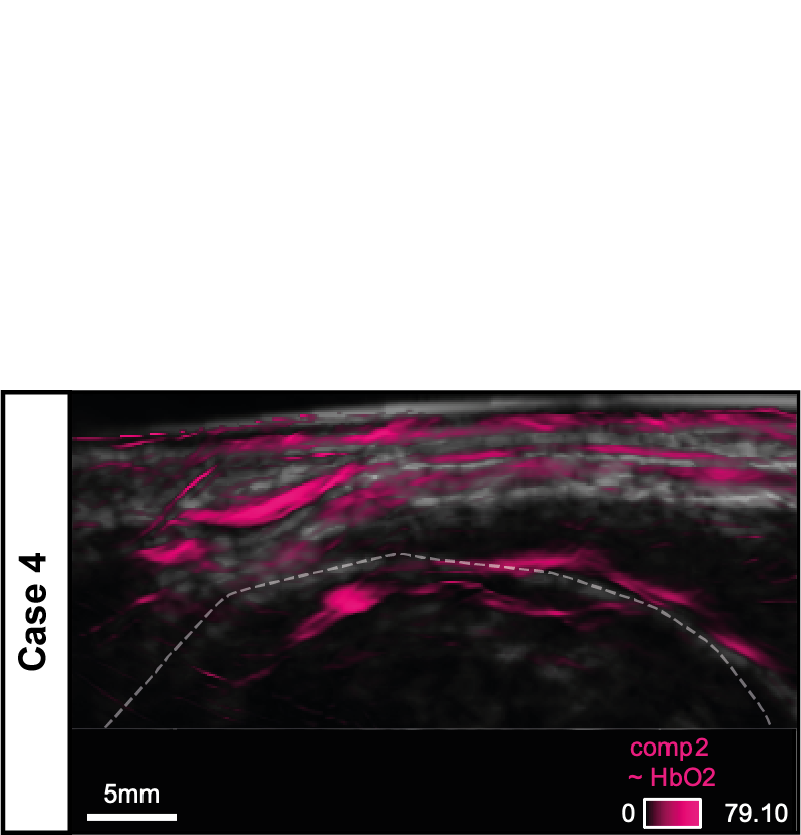


**Supplementary Figure 3. The HbO2 image of papillary thyroid carcinoma of Case 4.** MS-OPUS shows the HbO2 component similar to HbR component (see Figure 3, panel v). Because the spectra of the vessels determined in the MS-OPUS images is more similar to HbR than HbO2, we display comp 4 (~HbR) in the manuscript.


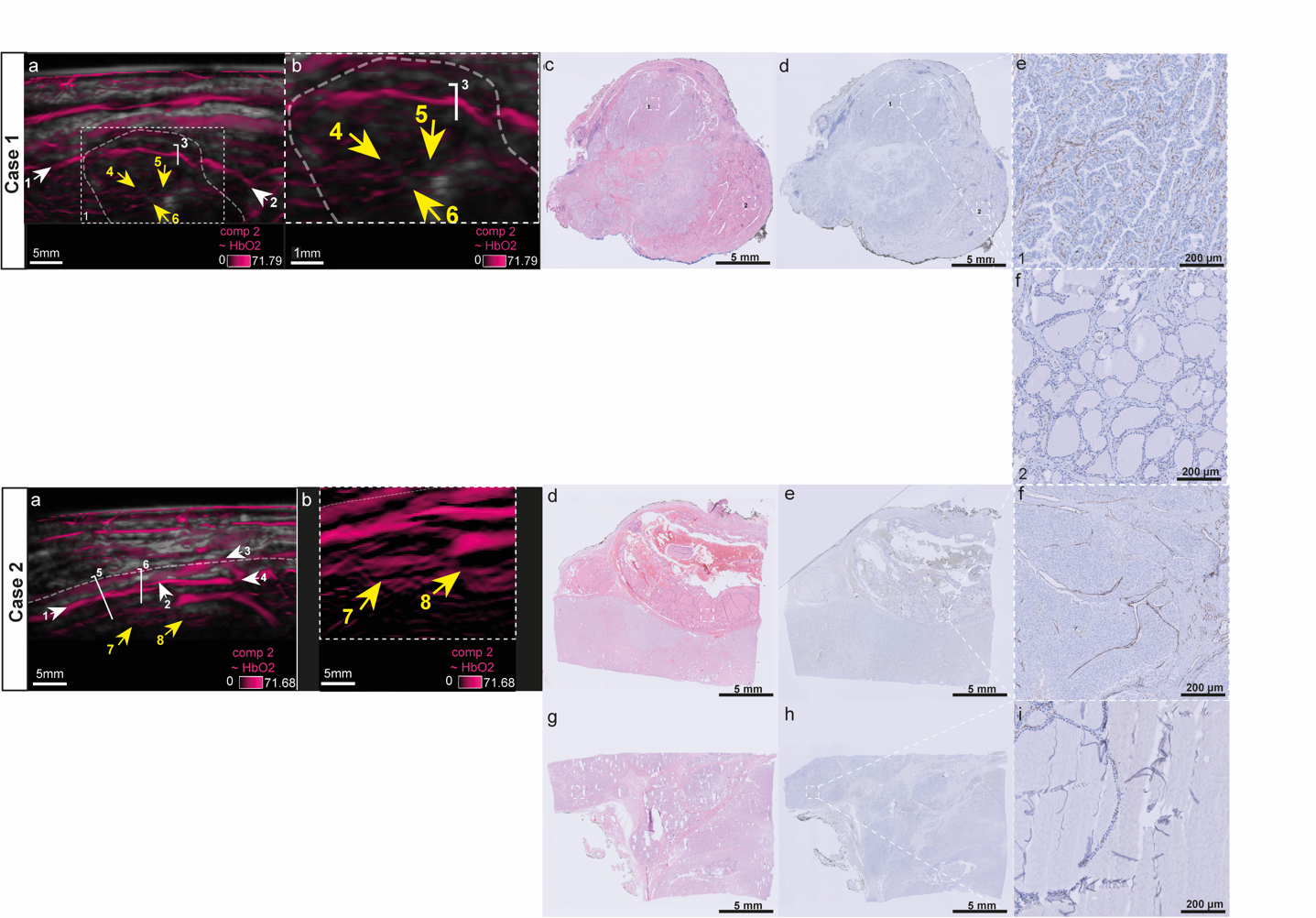


**Supplementary Figure 4. Case 1 and case 2 microvascularity in the thyroid nodule**. MS-OPUS (a) shows rich microvascularity in the thyroid nodule (yellow arrows 4, 5 and 6). Panel b is a more detailed image of the microvascularity. Immunohistochemistry with anti-CD31 antibody (d) shows higher microvascularity in the malignant thyroid nodule (e, quantified as 5+) than in the healthy thyroid tissue (f, quantified as 1+). MS-OPUS of case 2 (a) shows rich microvascularity in the thyroid nodule (yellow arrows 7 and 8). Panel b is a more detailed image of the microvascularity. Immunohistochemistry with anti-CD31 antibody shows higher microvascularity in the malignant thyroid nodule (e, f, quantified as 3+) than in the healthy thyroid tissue (h, i, quantified as 1+). Hematoxylin and Eosin (c, d, g) stained tissue sections are added for correlation.


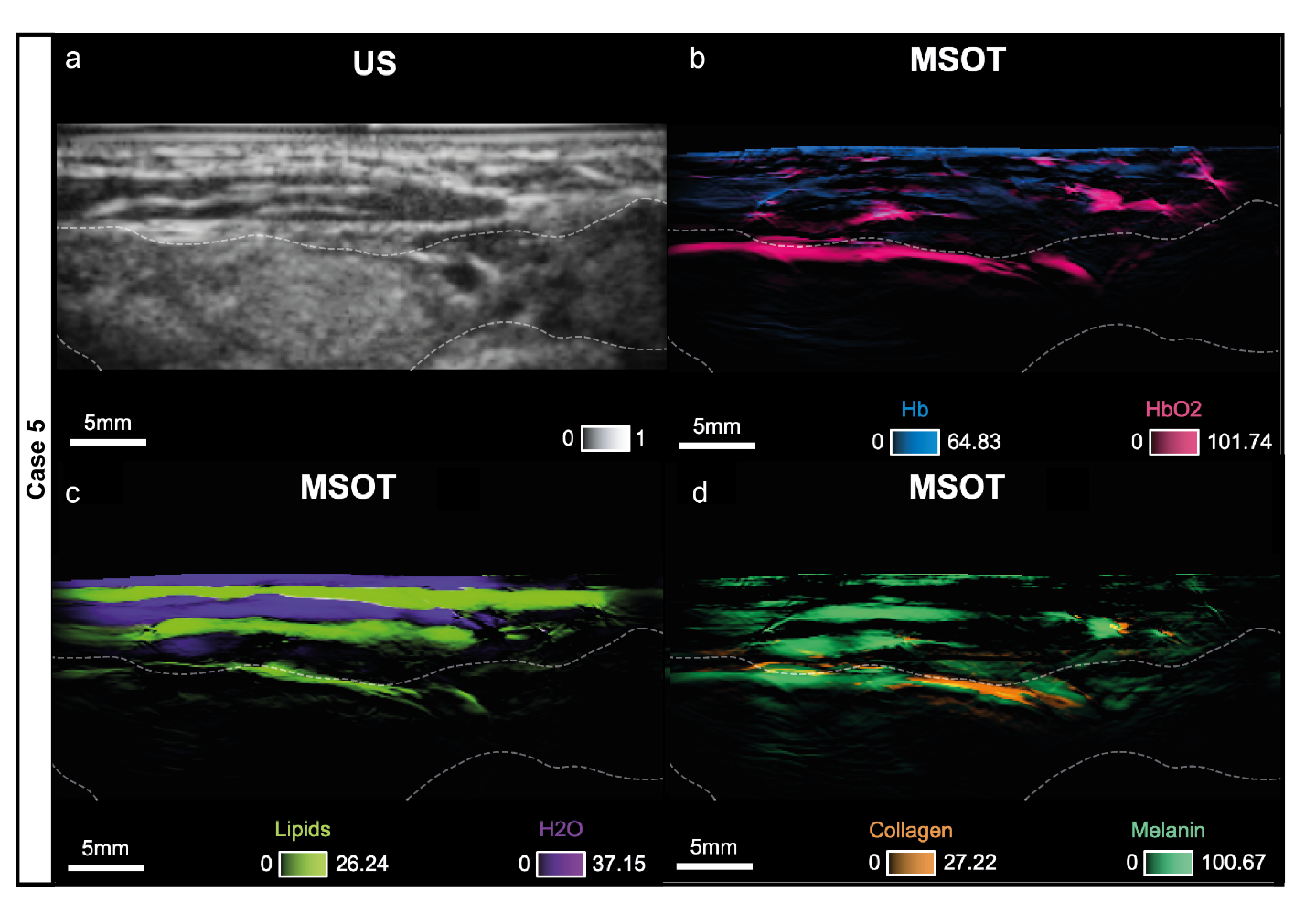


**Supplementary Figure 5. The linear unmixed components and the US image for Case 5 (multinodular goiter)**). The thyroid gland is delineated in white. We employed linear unmixing with Hb, HbO2, Lipids, H2O, Collagen and Melanin on the data set and showcase the results for Case 5 here for comparison with the blind unmixing results we display in the manuscript.

**
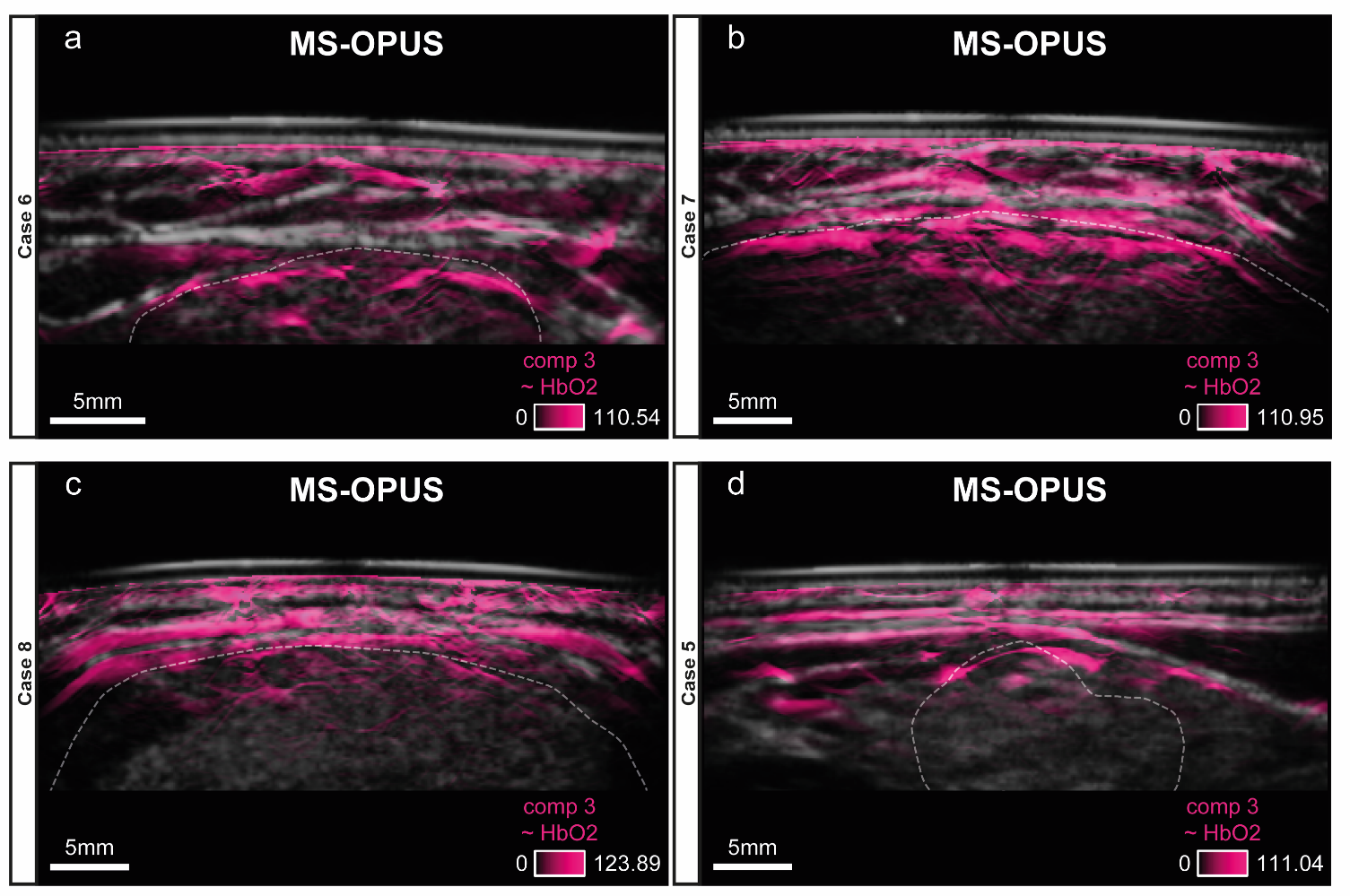
**

**Supplementary Figure 6. Additional scan for the remaining 3 cased (panel a-c) and Case 5 (panel d).** All cases are benign thyroid nodules.


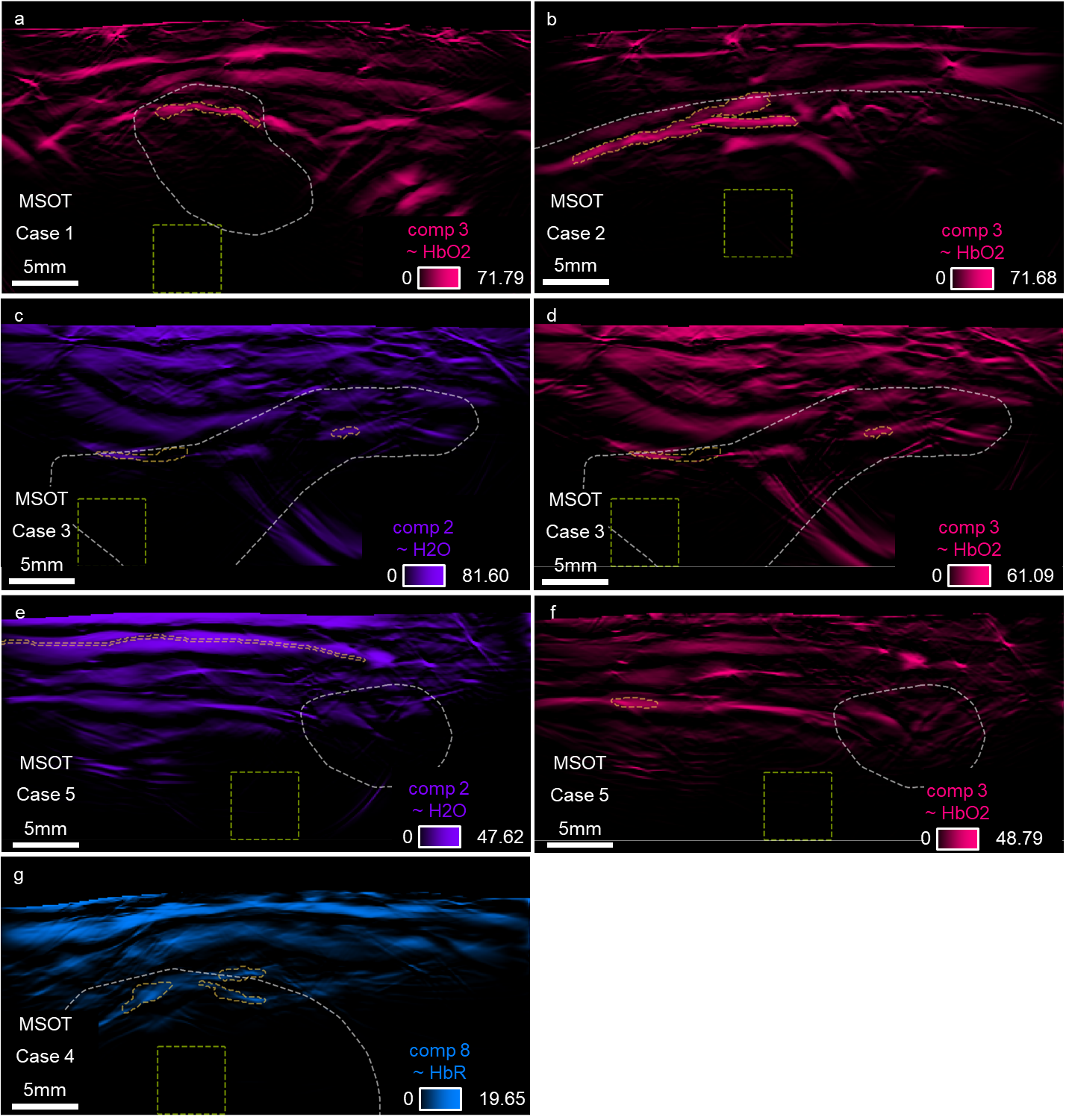


**Supplementary Figure 7. MSOT images of Cases 1-5 with all ROIs segmentations after blind unmixing.** Cases 1 (a), 2 (b) and 4 (G) show a thyroid carcinoma, Case 3 (c,d) shows a healthy thyroid and Case 5 (e,f) displays a the index nodule of a patient with multinodular goiter which are delineated in white respectively. For all Cases we determined mean spectra over an ROI which are delineated in yellow. For Cases 1-4 (Panels a,b,c,d,g) prominent vessels are displayed, and for Case 5 it is the region of the muscle fascia (Panel e) and a section of the prominent capsule of the thyroid gland (Panel f). For all regions delineated in yellow, we also computed the contrast resolution against image background delineated in green to quantify the image contrast.


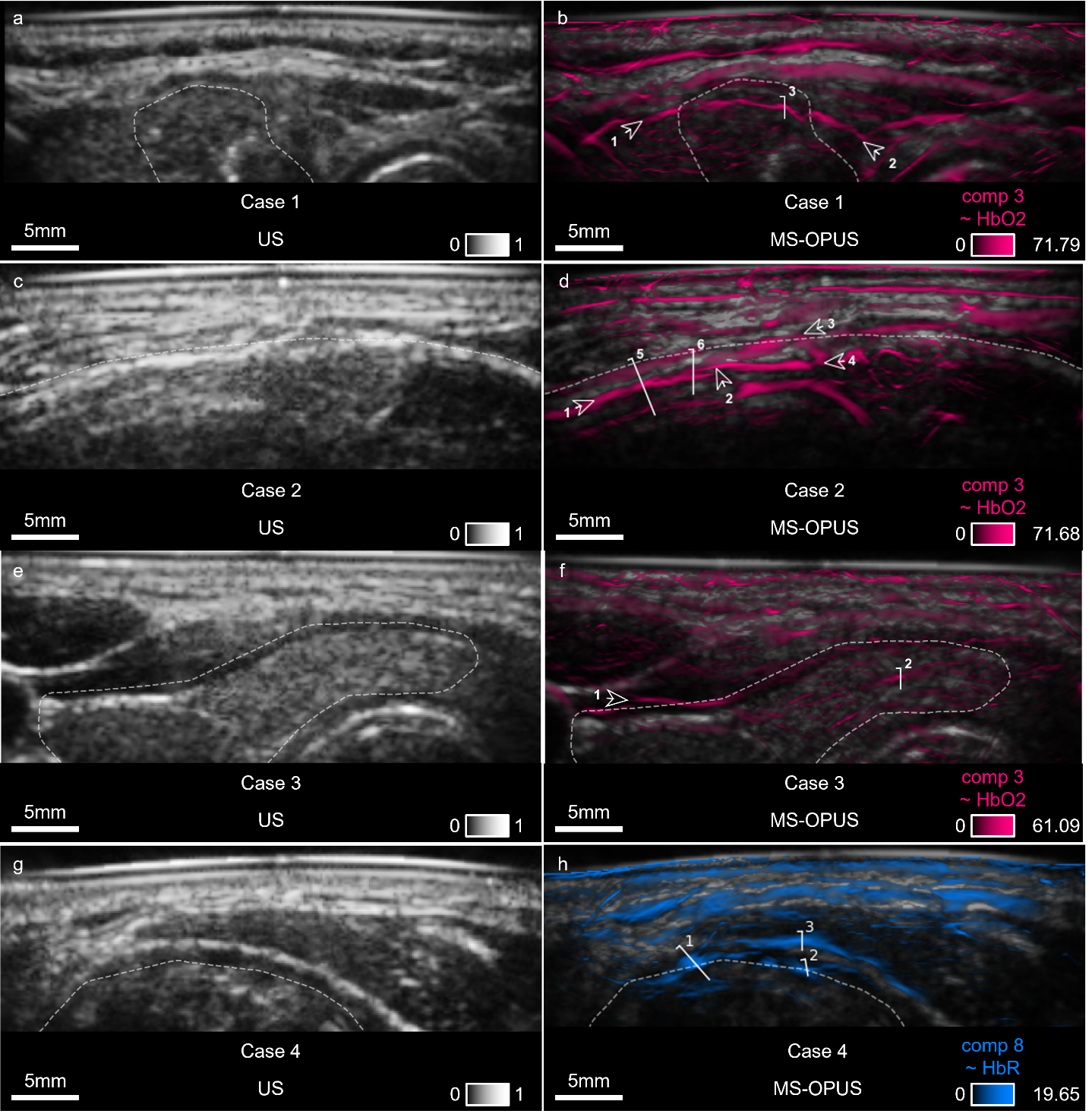


**Supplementary Figure 8. US and MS-OPUS images of Cases 1-4.** For Cases 1-4 we measured diameters of prominent vessels (Panel b: line 3, Panel d: lines 5,6, Panel f, line 2, Panel h: lines 1,2,3) and compared them to histopathology. None of the vessels can be detected in the US image (Panels a,c,e,g) alone substantiating the complementary information on vascularity provided by MSOT.
